# Average Lifetime Work Exposures and Immune Function Among Older Adults

**DOI:** 10.1093/geroni/igaf122.4075

**Published:** 2025-12-31

**Authors:** Rebekah Carpenter, Dawn Carr, Grace Noppert

**Affiliations:** Florida State University, Tallahassee, Florida, United States; Florida State University, Tallahassee, Florida, United States; University of Michigan, Ann Arbor, Michigan, United States

## Abstract

Overall immune health and functioning plays a critical role in the development of aging-related chronic conditions and diseases over the life course. Recent research shows that differences in immune function in later life are socially patterned and shaped by lifetime exposures. However, limited attention has been given to the potential impact of work-related factors on later-life immune health. Given that the average adult spends approximately one-third of their life working, occupational conditions may have long-term influences on immune function as people age over time. We use a new dataset that leverages occupation-level work exposures from the Occupational Information Network (O*NET), individual-level work history data, and individual immune function data from the Health and Retirement Study (HRS). Ordinary Least Square (OLS) regression models were assessed to evaluate associations between three domains of lifetime work exposures and eight indicators (e.g., Cytomegalovirus IgG antibody levels) of immune function after age 65. We find that we find that multiple stress- (e.g., job demands), physical- (e.g. frequency of bending and twisting the body), and environmental-related (e.g., exposure to contaminants) average lifetime work exposures are associated with later life immune health. We also find that the association between some work exposures (e.g., decision freedom) and immune function are dependent on sex, suggesting that for some occupational exposures, the same level of exposure influences immune function outcomes differently for men and women. Based on these findings, we determine that work exposures may be an important social determinant of immune function as people age over the life course

